# Adverse event using Medtronic NIM^™^ EMG endotracheal tube on a patient receiving anesthesia for hemithyroidectomy: a case report

**DOI:** 10.1186/s12871-022-01762-x

**Published:** 2022-07-13

**Authors:** Emilee Carpenter, Lorraine Norris, Myriam Beniamin

**Affiliations:** 1 State University of New York Upstate Medical University, College of Medicine, Syracuse, NY US; 2 Department of Anesthesiology, Canton Potsdam Hospital, Potsdam, NY US

**Keywords:** Anesthesia, Adverse events, Case report, EMG, Endotracheal tube

## Abstract

**Background:**

The neural integrity monitor (NIM) electromyogram (EMG) endotracheal (ET) tube is a widely used device to monitor neural response through muscle activity. It is helpful in surgical procedures with high risk of damaging delicate structures in the head and neck. This case provides a thorough analysis of an adverse event that was encountered in the operating room, which others can hopefully learn from.

**Case presentation:**

We are reporting a case in which a patient undergoing hemithyroidectomy had experienced an adverse event using the Medtronic NIM^™^ EMG endotracheal tube. After successful induction and intubation, confirming the proper positioning of the electrode wires was necessary before the incision could be made. Upon reexamination, the patient suddenly became difficult to ventilate with increased peak airway pressure, decreased tidal volume, and end tidal CO2. This episode lasted approximately 15 min and the patient’s condition remained stable despite low tidal volumes. The problem was unexpectedly resolved upon deflation of the cuff of the ET tube.

**Conclusions:**

There are several similar reports of these endotracheal tubes causing obstruction, especially those in which overinflation of the cuff caused cuff herniation and blockage of the Murphy eye and the bevel. It is currently believed that the design of this tube allowed for the obstruction to occur. The patient’s short body habitus may have also been a small contributing factor. The distance that the electrodes must sit within the vocal cords to the tip of the bevel is longer in this type of ET tube compared with a standard ET tube. The distance from the true vocal cords to where the cuff sits in the trachea is also greater in this model NIM EMG tube. There was no confirmation of the exact obstructive process that took place, however, confirming the tube and cuff positioning would have been optimal.

## Background

The neural integrity monitor (NIM) electromyogram (EMG) endotracheal tube is commonly utilized in head and neck procedures [1]. Such procedures include but are not limited to hemithyroidectomy, thyroidectomy, parathyroidectomy, neck dissection, carotid endarterectomy, and partial laryngectomy [11]. Electromyography monitors muscle activity during a surgery that poses risk to nearby structures such as cranial or spinal nerves [4]. The tube contains stainless-steel electrodes that must sit on each vocal cord to monitor their activity. Color indicators on the tube aid providers in the particular placement of these electrodes. The output is an audiological signal when the muscle is stimulated that can be detected easily by the surgeon.

Here we describe a case in which an unexpected outcome from the use of the Medtronic NIM^™^ EMG endotracheal tube on a patient receiving anesthesia for a partial thyroidectomy had occurred. While similar cases exist, this case is unique in that it offers a full in-depth analysis of the situation, possible explanations, and future recommendations.

## Case Presentation

This case describes a middle-aged female who presented to the ambulatory surgery center for right sided hemithyroidectomy. Pre-operatively, a full history and physical exam were obtained. The patient had received anesthesia in the past without complications. The patient is a nonsmoker. On physical exam, the patient was well-appearing and in no acute distress. Vital signs within normal limits. The patient’s height, weight, and BMI are 155 cm, 78 kg, and 32.5, respectively.

Airway exam: mallampati: 2, Thyromental distance: 2.5 Fingerbreadths.

Neck exam: full range of motion, able to protrude mandible, normal neck circumference, upper denture was present.

The patient was classified by the American Society of Anesthesiologists (ASA) as a risk of 3. The anesthesia plan was general-INH anesthesia with endotracheal intubation using the Medtronic NIM^™^ Standard Reinforced EMG Endotracheal tube.

Intubation was completed with the Medtronic NIM^™^ EMG ET tube size 7.0 was successful using a Glidescope. Airway assessment with the Glidescope showed a grade 1 view. The red wire was located on the right side of the cords and the blue wire on the left, according to the manufacturer’s protocol. The tube was taped at 22 cm. The cuff was inflated with 10mL of room air. The patient was ventilated using volume control on the anesthesia machine and maintained with sevoflurane. Following surgical positioning with neck extension and prior to incision, it was necessary that the surgeon confirmed the proper placement of this endotracheal tube, such that the EMG leads were in direct contact with the patient’s true vocal cords. As tube placement confirmation was taking place, the surgeon extended the patient’s neck and inserted the Glidescope to view the electrodes touching the vocal cords. They were in the correct position, however, as this was taking place, the patient abruptly became difficult to ventilate. Very low tidal volume was noted on the anesthesia machine and higher pressures were required to ventilate. In addition, there was an absent End-Tidal Carbon Dioxide (EtCO2) reading on the monitor. The anesthesia machine was re-checked for leaks with none found. Patient received albuterol (2.5 mg nebulized) with no improvement. On auscultation of the chest, there was minimal breath sounds on either side and minimal chest rise on inspection. A suction tube was placed down the tube to rule out an obstructed tube by a mucous plug or other foreign body, however, the suction passed down easily with no feedback. It was then decided to exchange this particular ET tube to a standard endotracheal tube in hopes of improving ventilation. However, upon deflation of the cuff prior to removal of the tube, the patient’s tidal volumes had returned to normal limits. The cuff was reinflated and there were no subsequent issues with ventilation thereafter. This episode lasted approximately 15 min. During this time the patient’s oxygen saturation remained stable at 100%, dropping to 99% once. The EtCO2 readings were zero consistently throughout the episode and the tidal volumes were read around 10–20 mL. The patient had an uncomplicated post-operative period.

## Discussion and conclusions

During this adverse event, several differential problems were under investigation. The rapid onset and inability to ventilate after head manipulation led to a wide differential including right mainstem bronchus intubation, bronchospasm, anesthesia machine/tubing leak, displaced tube, tube blockage by a mucous plug, and an alternative obstructive process. Much of these differentials were ruled out by intra-operative testing and examination. This left only one option on the differential; an anatomical obstruction caused by the tube itself that corrected upon patient head movement and cuff repositioning. As the mechanics behind this differential were perplexing to the members of the team, it was decided to preserve the ET tube after the case had concluded for visual inspection.

**Fig. 1:**
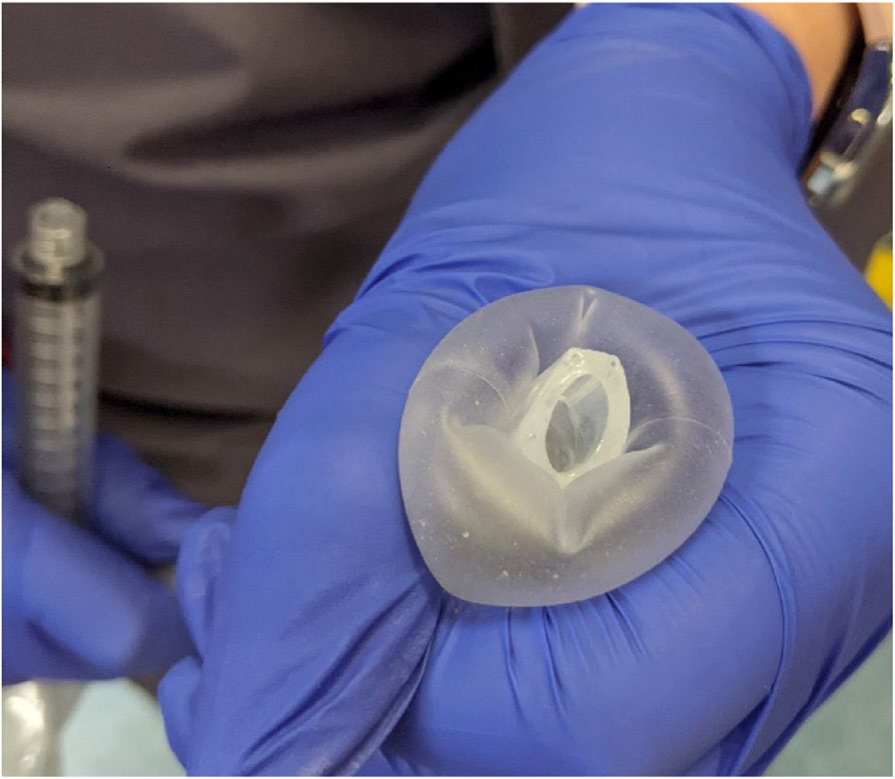
NIM EMG Endotracheal Tube 7.0 mm I.D. x 10.2 mm O.D. Cuff inflated and manually manipulated to extend to the end of the bevel and therefore obstructing the Murphey eye

On visual inspection, the ET tube was intact, the pilot balloon and cuff were not damaged and could hold an adequate amount of air. During the case, 10mL of air filled the cuff, although a much greater volume could be held by the cuff as it was rather compliant. When manipulating the cuff with some force, we could move it in such a way that the cuff enclosed the Murphy eye and could partially obstruct the bevel as seen in Fig. 1.

**Fig. 2:**
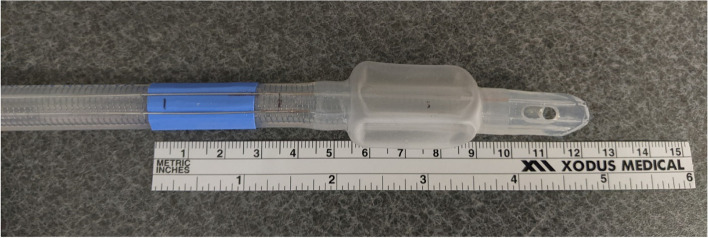
NIM EMG Endotracheal Tube 7.0 mm I.D. x 10.2 mm O.D. Length from top of the recommended vocal cord placement (blue) to the tip of the bevel

We compared this Medtronic NIM^™^ EMG ET tube to a standard ET tube of the same size. We noticed that the Murphy eye of the standard tube was slightly larger in size, measuring 16 mm x 8 mm compared to 10 mm x 5 mm. Additionally, the length from the bevel to where the tube sits on the vocal cords was much longer when compared to the standard ET tube. According to the manufacturer’s instructions of use, “the area of the tube, with contrasting color, must be in contact with the true vocal cords.” Additionally, the manufacturer recommends sizing the tube 1 size greater than what would typically be used for the patient’s size as to ensure proper contact between the electrodes and the true vocal cords. This distance, which can be appreciated in Fig. 2, is much longer than the standard ET tube. In a standard ET tube, the cuff typically sits just below the vocal cords, but the cuff must sit more distal to the cords in the NIM EMG tube due to the location of the exposed electrode wires, located only throughout the blue colored section.

The standard length of a trachea in a female of this age group is 9 cm ± 1 cm [12]. It has also been found that height and weight significantly influence the length of one’s airway. According to a study focused on lengths of the trachea and main bronchus in the Chinese Shanghai population, lengths of the trachea are significantly different between males and females. This study found that the mean trachea length for these females was 12.9 cm ± 1.23 cm in the age group of 50.82 yrs. ± 14.59 yrs. [3]. Our patient has a measured trachea length of 11.7 cm from vocal cords to carina and 11.1 cm from vocal cords to the mainstem bronchi as measured by radiographical imaging. This cannot exclude that a short-statured female, such as our patient could have a trachea length equal to or even shorter than the distance from the ideal vocal cord placement of the tube to the bevel as seen in Fig. 2. The tip of the bevel would sit approximately 9.5–12 cm distal to the trachea with proper electrode placement.

Additionally, upon neck extension, the distance from the upper incisor to the carina was found to increase by 1.99 cm. Much of this increased distance is attributed to area from the vocal cords to the sternal notch. Thus, ET tubes fixed at the mouth tend to ascend in the trachea upon extension of the neck [13]. What we can deduct from this data it is that in a short statured female, it is possible that the trachea length is shorter than the distance the endotracheal tube must travel. This also may partially explain why the patient’s condition improved when attempting to exchange the tube, as her head and neck were manipulated.

The optimal depth of NIM EMG endotracheal tubes was found to be 19.6 ± 1.0 cm in women and 20.6 ± 0.97 cm in men (*p* < 0.01). The patient’s height was also directly related to the depth of the tube [5]. Our patient had a height of 154.94 cm, and the tube was inserted and taped at the lip at 22 cm.

We propose that the distance from the vocal cords to the bevel of the Medtronic NIM^™^ EMG ET tube that was used in this patient was inappropriately long. During manipulation of the airway to ensure proper placement of the EMG wires, the bevel could have been pressed upon the patient’s carina, partially obstructing the end. At the same time, the flexibility of the cuff made it such that the right and left mainstem bronchi were occluded and/or the Murphy eye was blocked. This resulted in an obstructed picture with very low tidal volumes, exceedingly large pressure needed to ventilate, absent EtCO2, and no breath sounds. CO2 was accumulating due to inability to overcome the dead space ventilation. This theory also explains why the patient’s condition improved upon cuff deflation, which was counterintuitive. When the cuff was inflated for the final time, it had repositioned itself to sit higher up in the trachea, rather that obstructing both bronchi. An additional theory as to what happened during this incident is that the cuff could have blocked both the Murphy eye and the end of the bevel, such that almost no airflow could get through the tube. We believe this is less likely because the amount of force required to displace the cuff that much would have only been achieved by pulling back on the tube while the cuff was still inflated. When the patient’s neck was extended to get a view of the tube, the tube could have ascended the trachea while the cuff remained in place, thus creating a blockage.

Recommendations from the manufacturer’s instructions of use state to avoid overinflation of this particular cuff. The ideal pressure inside the cuff should be at or below 25 mmH2O. It is also recommended to measure this pressure with an ET tube manometer. At our facility, we did not have access to one of these manometers. Therefore, we inflated the cuff with 10mL of room air. Medtronic does not recommend estimating the cuff pressure by feel or resistance or by volume alone [10]. In addition, they also recommend monitoring cuff pressure throughout the procedure. The concern mentioned in the instruction manual was overinflation of the cuff. Since the pressure in the cuff was not measured, we cannot exclude this as a contributing factor in the adverse event that took place. However, we noticed that it was when the cuff had less pressure and thus more flaccid, it could stretch over the end of the tube, which would support our theory above.

There are several adverse event reports that exist with this same endotracheal tube. The Food and Drug Administration have recognized a number of these events, some of which are detailed below. An adverse event report from 2021 described a scenario in which a health care provider reported that the cuff self-herniated over the Murphy eye upon inflation. The tube was unable to be ventilated as the bevel was resting against the trachea wall. After deflation and reinflation, the same event occurred. Observations made by this medical team showed that the material of the cuff seemed thinner and softer when compared to the cuffs of other tubes. Additionally, the Murphy’s eye appeared smaller than in other models [7]. Similar observations were made in this case. Another report from 2021 described the same incident of cuff herniation over Murphy’s eye. The obstruction in this case was confirmed with an endobronchial fiber optic device [6]. A similar case report published in 2012 described a scenario in which the same Medtronic NIM^™^ EMG endotracheal tube size 7 mm was used under general anesthesia for a total thyroidectomy. In this adverse event, excessive air was added to the cuff and during the surgery, ventilatory peak pressure had increased while the oxygen saturation had decreased. The manufacturer notes that there is reasonable evidence to suggest that the product had malfunctioned. Overinflation may cause cuff deformation, deflation, or rupture resulting in tube blockage and/ or tracheal damage [8]. As many as 5 additional adverse event reports have been filed with the Food and Drug Administration which describe similar obstruction-like problems with the device [9].

Silicone-based endotracheal tubes such as the Medtronic NIM^™^ EMG tube may be more prone to cuff herniation causing airway obstruction, likely due to the elasticity of the cuff. At higher cuff pressures, the cuff may inflate asymmetrically and obstruct the distal end of the tube [2]. Since we had inflated the cuff with only 10 mL of air, we do not believe that overinflation was the problem in our case.

Overall, we believe that the patient’s short body habitus in conjunction with the longer distance from bevel to electrodes, and the anatomical differences between this NIM EMG tube compared with standard tubes caused and obstruction in the patient’s airway. The obstruction was likely due to the bevel tip lodged against the airway wall and the endotracheal tube cuff possibly obstructing either the main stem bronchi, the Murphy eye, or both. Even with the electrode wires placed in the correct location, the anatomy and length of this tube still allowed for and obstruction to take place. In the future, we may recommend confirming the cause of the obstruction with fiber optic technology. At the time of the event, airway management and patient safety took precedent over diagnostics. In addition, we would recommend ensuring adequate pressure in the ET tube cuff to ensure neither overinflation nor underinflation. Lastly, manufacturing these tubes to account for varying trachea lengths amongst the population, and/or designing the tube such that the cuff is located more proximally could prevent this from occurring in the future. 

Limitations of this report include the restricted time to diagnose the problem on the operating room as all attention was focused on preserving the outcome of the patient. In addition, having access to more diagnostic equipment would have been useful.

## Data Availability

All data generated or analyzed during this study are included in this published article.

## Abbreviations

ASA: American society of anesthesiologists
CO2: Carbon dioxide
EMG: Electromyography
ET: Endotracheal tube
EtCO2: End-tidal carbon dioxide
I.D: Inner-diameter
NIM: Neural integrity monitor
O.D: Outer-diameter
SpO2: Oxygen saturation
